# Metabolic fingerprinting of chemotherapy-resistant prostate cancer stem cells. An untargeted metabolomic approach by liquid chromatography-mass spectrometry

**DOI:** 10.3389/fcell.2022.1005675

**Published:** 2022-10-17

**Authors:** Alicia Bort, Belén G. Sánchez, Carlos León, Leonor Nozal, José M. Mora-Rodríguez, Florentina Castro, Antonio L. Crego, Inés Díaz-Laviada

**Affiliations:** ^1^ Yale University School of Medicine, Vascular Biology and Therapeutics Program, New Haven, CT, United states; ^2^ Alcala University, School of Medicine, Department of Systems Biology and Research Institute in Chemistry “Andrés M. Del Río” (IQAR), Madrid, Spain; ^3^ Carlos III University, Department of Bioengineering and Aerospatial Engineering, Madrid, Spain; ^4^ Alcala University and General Foundation of Alcalá University, Center of Applied Chemistry and Biotechnology, Madrid, Spain; ^5^ Alcala University, Department of Analytical Chemistry, Physical Chemistry and Chemical Engineering, Madrid, Spain

**Keywords:** prostate cancer, cancer chemoresistance, fatty acid oxidation, cancer stem cells, untargeted metabolomic, liquid chromatography-mass spectrometry

## Abstract

Chemoresistance is one of the most important challenges in cancer therapy. The presence of cancer stem cells within the tumor may contribute to chemotherapy resistance since these cells express high levels of extrusion pumps and xenobiotic metabolizing enzymes that inactivate the therapeutic drug. Despite the recent advances in cancer cell metabolism adaptations, little is known about the metabolic adaptations of the cancer stem cells resistant to chemotherapy. In this study, we have undertaken an untargeted metabolomic analysis by liquid chromatography–high-resolution spectrometry combined with cytotoxicity assay, western blot, quantitative real-time polymerase chain reaction (qPCR), and fatty acid oxidation in a prostate cancer cell line resistant to the antiandrogen 2-hydroxiflutamide with features of cancer stem cells, compared to its parental androgen-sensitive cell line. Metabolic fingerprinting revealed 106 out of the 850 metabolites in ESI+ and 67 out of 446 in ESI- with significant differences between the sensitive and the resistant cell lines. Pathway analysis performed with the unequivocally identified metabolites, revealed changes in pathways involved in energy metabolism as well as posttranscriptional regulation. Validation by enzyme expression analysis indicated that the chemotherapy-resistant prostate cancer stem cells were metabolically dormant with decreased fatty acid oxidation, methionine metabolism and ADP-ribosylation. Our results shed light on the pathways underlying the entry of cancer cells into dormancy that might contribute to the mechanisms of drug resistance.

## 1 Introduction

Prostate cancer (PCa) is the second leading cause of cancer-related death in men with an annual incidence of 268500 cases (which supposes 27% of all cancer types in men) (Siegel et al., 2022). According to data from the Spanish Society of Medical Oncology (SEOM), PCa was the most frequent and prevalent malignant neoplasm in Spain in 2020 (Cassinello et al., 2018; Gonzalez Del Alba et al., 2021). Despite the recent highly developed treatments, the advanced disease, characterized by androgen-independent growth, experiments 4%–6% annual increase (Siegel et al., 2022). Once prostate cancer progresses to become castration-resistant prostate cancer (CRPC), therapy is based on androgen receptor inhibition and/or antimitotic taxane chemotherapy which improve survival in patients (Menges et al., 2022). However, despite the initial benefits of such therapy prostate cancer, more often than desired, becomes resistant to chemotherapy and then, the effect of chemotherapeutic treatments is hampered and therapeutic options are limited (Lohiya et al., 2016). Currently, the development of chemoresistance represents one of the greatest challenges in cancer treatment. Resistance to treatments may be caused by the presence of cancer stem cells (CSCs), a subset of cells within the tumor with long-term replicative potential, self-renewal, and pluripotency abilities.

It has been described that prostate cancer stem cells are resistant to most standard therapies and constitute a source for the clonal expansion of cancer cells that is thought to be responsible for tumor recurrence (Leao et al., 2017; Skvortsov et al., 2018). CSCs sustain tumor growth by reactivating signaling pathways and adaptive metabolic networks that induce the proliferation of surrounding cells and stem cell plasticity. CSCs sense energy demand and nutrient supply, and display different metabolic pathways compared to the rest of tumor cells. Metabolic reprogramming is a hallmark of cancer and allows tumor cells to meet the increased energy demands required for rapid cell growth, invasion, and metastasis. Notably, metabolic reprogramming prevents cancer cells from chemotherapy-induced death contributing to cancer drug resistance. This is especially relevant in prostate cancer in which dysregulated lipid signaling fuels growth and obesity and has been described as a risk factor with a strong correlation with advanced or lethal prostate cancer (Pernar et al., 2018). Accumulated evidence suggests that metabolic adaptations in CSCs are different from that in bulk cancer cells and quite similar to that in normal tissue stem cells (Kim, 2019). Moreover, the altered differentiation signals that induce CSC may be controlled by metabolic events that take part in the regulation of stem cell fate (Menendez and Alarcon, 2014). For instance, some metabolites can regulate epigenetic changes, including histone methylation and acetylation involved in the control of gene expression (Shyh-Chang and Ng, 2017). However, the metabolic signature of CSC resistant to chemotherapy remains elusive.

Metabolomics aims to detect, identify and quantify all metabolites in a biological sample, using chemometric and statistics tools in order to compare the metabolic fingerprints or metabolite profiles of different physiological conditions which depend on genetic and environmental factors (Dieme et al., 2017). Metabolomics provides information about the biochemical state or metabolome of biological system under certain conditions. This methodology allows to know the low molecular weight compounds present in cells, tissues or biological fluids, which are involved in different metabolic pathways necessary for the maintenance, growth and normal functioning of a cell (Fiehn, 2002; Zhang et al., 2014; Xu et al., 2021). Furthermore, the metabolic profile changes over time, and at different points in a disease. Recently, metabolomics has started to be applied in CSC research, because it can show the energetic status, cell proliferation and fitness, and stem cell fate choices such as self-renewal *versus* differentiation (Sun et al., 2019) as well as the cross-talk between the cell and its environment (Bispo et al., 2021).

The detection of metabolites in cells, tissues or biofluids is usually carried out using advanced profiling analytical techniques like Nuclear Magnetic Resonance (NMR) spectroscopy (Sciarra et al., 2011; Lima et al., 2018; Vandergrift et al., 2018) or Mass Spectrometry (MS) (Zang et al., 2014; Segers et al., 2019; Sun et al., 2022) which have been successfully applied to prostate cancer study. In general, NMR spectroscopy and MS, particularly Liquid Chromatography–High-Resolution Mass Spectrometry (LC-HRMS), are the two most important analytical platforms used in metabolomic studies. Both techniques provide large amounts of data, which require statistical analysis to extract relevant and robust information on metabolic response. One of the main challenges is the complexity of any metabolome, and the “untargeted strategy” is the most adequate to establish the complete profile of the metabolites present (“metabolic fingerprinting”) in a biological system (Alonso et al., 2015). The advantage of MS over NMR is the higher sensitivity to detect metabolites at much lower concentrations and the more suitability for high throughput methods. In addition, in recent years, the LC-HRMS technique has been increasingly used due to its robustness and resolution capacity. The high resolution in the determination of the mass/charge ratio (m/z) of the detected ions together with the specialized databases in MS, allow to search for unknown compounds and the tentative identification of the metabolites present. Thus, today LC-HRMS is the best analytical tool to carry out metabolomics studies (Jayaraman et al., 2018; Martin-Blazquez et al., 2019; Pinto et al., 2020).

Here, we use a metabolomic approach using LC-HRMS, to understand the adaptive response linked to chemotherapy resistance using a model of cancer cells resistant to therapy that displayed features of CSCs. Our combined analysis of the metabolic fingerprinting and enzyme analysis identified that drug-resistant cells have a low metabolic rate characterized by low ADP-ribosylation and methylation as well as low mitochondrial fatty acid β-oxidation. The identified metabolic pathways in prostate-resistant cells will allow the development of effective therapeutic strategies that target the mechanisms involved in the acquisition of drug resistance and deserves further investigation.

## 2 Materials and methods

### 2.1 Cell culture

The human prostate cancer cell line LNCaP was obtained from American Type Culture Collection (ATCC CRL-1740, Rockville, MD, United States) and cultured in RPMI-1640/10%FBS plus 100 IU/ml penicillin G sodium, 100 g/ml streptomycin sulfate and 0.25 g/ml amphotericin B (Invitrogen, Paisley, United Kingdom). To generate the prostate cancer resistant Cell line, LNCaP cells were cultured for 6 months in RPMI-1640/10%FBS with a stepwise concentration increase of the antiandrogen 2-hydroxyflutamide, starting at 0.1 µm. When the cells were capable of growing and reaching appropriate confluency at this concentration, the cells were passaged and 2-hydroxyflutamide concentration was increased in 0.1 µM. When cells were able to grow in the presence of 2 µm 2-hydroxyflutamide, were routinely maintained in such concentration and renamed LN-Flu.

### 2.2 Sample preparation for metabolome analysis and extraction of intracellular metabolites

LNCaP or LN-Flu cells (20×10^6^) were seeded in each 100 mm culture dish and cultured for 48 h. When cells were 80% confluent, were washed in PBS at 4°C and frozen at -80°C. Then cells were harvested and incubated for 20 min at 4°C in lysis buffer (50 mM Tris pH 7.4, 0.8 M NaCl, 5 mM MgCl2, 0.1% Triton X-100, containing protease inhibitor and phosphatase inhibitor cocktail) (Roche, Diagnostics; Mannheim, Germany). Cell extracts were centrifuged at 10000 g at 4°C. The supernatant was concentrated by centrifugation at 10000g at 4°C in Amicon ultra-0.5, ultracel filters (Amicon, Miami, Florida, United States). Samples were stored at -80°C until their analysis.

### 2.3 Chemicals and standards

HPLC grade acetonitrile, MS grade formic acid and ammonium acetate for LC-MS (Carlo Erba Reagents Srl, Chaussée du Vexin, France), water obtained from a Milli-Q system (MilliPore, Bedford, MA), and acetic acid Optima LC-MS (Thermo Fischer Scientific Inc., Madrid, Spain) were used in the preparation of mobile phases. The list of standards included in this study is shown in Supporting Information Supplementary Table S1.

### 2.4 Liquid chromatography–high resolution mass spectrometry analysis

Prior to LC-MS analysis, the extracted samples (*n* = 20 LNCaP and *n* = 20 LN-Flu, different cell cultures) were thawed, homogenized in vortex for 2 min and submitted to an ultrafiltration process with 3 kDa ultrafiltration membranes (Merck KGaA, Darmstadt, Germany). 500 µl of each sample were introduced into the ultrafiltration membrane and a centrifugation step was carried out at 4,000 g at 4°C for 40 min. Finally, each ultrafiltered sample was divided into two aliquots of 150 µl to be analyzed by LC-MS (one used in positive polarity and the other in negative), one aliquot of 100 µl to constitute the total quality control (QCT) sample that includes all the study samples, and one aliquot of 50 µl to constitute the sample of quality control of each study group (QCG1 and QCG2, LNCaP group and LN-Flu group respectively). In the case of all QCs, 20 µl of warfarin standard at 100 ng/ml was added for the internal control of the LC-MS system (retention time and mass accuracy of the warfarin). These QCs were injected every eight samples to monitor the performance, stability, and reproducibility of the LC-MS method throughout the analysis sequence. Several blanks and QCTs were injected at the beginning of the sequence to ensure good repeatability. Sample injection was randomized.

Untargeted metabolomic analysis of all samples was performed using a Thermo Scientific Dionex Ultimate 3,000 series Ultrahigh Performance Liquid Chromatograph (Waltham, Massachusetts, United States) coupled to Q-Exactive hybrid Quadrupole-Orbitrap mass spectrometer from Thermo Scientific (Waltham, Massachusetts, United States) equipped with an orthogonal electrospray ionization (ESI) source and operating in both positive and negative ion mode.

The reversed-phase HPLC method using a column Atlantis (100 mm × 2.1 mm, 1.6 µm porous particle) from Waters (Madrid, Spain) with its respective column guard, consisted of a linear binary gradient in three steps: 0% of B for 2 min of 0%–100% of B in 10 min, and 100% of B for 2 min. After analysis, the column was re-equilibrated for 5 min using the initial solvent composition. The flow rate was 0.4 ml/min, the injected volume was 5 µl and the column was kept at 40°C during the analytical sequence. The mobile phase in positive polarity was water (eluent A) and 90% (v/v) acetonitrile (eluent B), both with 0.1% (v/v) formic acid. In polarity negative were 5 mM de ammonium acetate (eluent A) at pH 5 adjusted with acetic acid at 12% (v/v) and 90% (v/v) acetonitrile (eluent B) with 5 mM de ammonium acetate at pH 5 adjusted with acetic acid at 12% (v/v). The mobile phases composed of de ammonium acetate were filtered using 45 µm nylon membrane filters from Merck KGaA (Darmstadt, Germany).

Mass spectrometer operated using full scan mode with a mass range of 70–2000 m/z, at a resolution of 140,000, a maximum injection time (IT) and AGC target of 480 ms and 1x10^6^, respectively. Furthermore, in order to carry out the confirmation of the detected compounds, a not target MS/MS method was applied with following parameters, resolution of 17,500, maximum IT and AGC target of 50 ms and 1x105, three normalized collision energies of 10, 20 and 30 V to have an average spectrum of the three energies, and an isolation window of 2.0 m/z. The source parameters were as follows: sheath gas flow rate, 60 arbitrary units; auxiliary gas flow rate, 30 arbitrary units; sweep gas flow rate, 0 units; spray voltage, 3.0 kV; capillary temperature, 280°C; vaporizer temperature, 400°C; and s-lens, 50. Data acquisition was performed by TraceFinder software 4.1 from Thermo Fischer.

### 2.5 Data handling and metabolite annotation

An untargeted data analysis strategy was used to obtain the maximum amount of information from the data acquired with the LC-HRMS method, including identification, grouping of features and statistical analysis. These features are groups of m/z signals that form an independent molecular entity, including molecular ions, adducts and isotopic ions, and they represent all the compounds or metabolites present in the analyzed samples.

All raw spectra were extracted and analyzed in centroid mode with a threshold of 1x10^5^ intensity units using the Compound Discoverer software (version 3.2.) from Thermo Fischer, in order to obtain a list of peak areas, retention times, and accurate mass to charge ratios (m/z). Peak preprocessing settings are as follow: Minimum peak intensity: 500.000 units; mass tolerance: 5ppm; S/N threshold: 3; retention time tolerance: 0.2 min. Possible unspecific or noise signals (S/N > 3) were removed while adducts of the same metabolite were grouped. This resulted in a list of features, each of which was identified with a unique ID. Filtering of the data was then carried out to ensure ions with a high quality: 1) peaks below 3x intensity from the blanks were removed, 2) peaks not found in at least a 80% of the samples belonging to the same group and 3) with a high variability within the same group (CV > 30% in the QCs and CV > 50% in the samples) were also filtered. Resulting data was normalized to the sum to minimize technical variation and Pareto scaling was applied to the metabolite intensities.

The resulting output data table of high-quality time-aligned detected features or metabolites, with their corresponding retention time, m/z and peak area obtained for each sample, were submitted to statistical analysis.

First, a Principal Component Analysis (PCA) was used to study variability among samples and features and for outlier detection. Once all possible sample outliers were removed, the Shapiro Wilk test was used to verify the normal distribution of the data. Afterwards, a *t*-test with the Benjamini-Hochberg False Discovery Rate (FDR) multiple testing correction was used for the level of significance of the tests. This significance is represented in a Volcano plot to evaluate features significantly different between group comparisons (FDR <0.05) and with a relevant Fold Change (FC) with values >2 or <1/2. The Metaboanalyst software (version 4.0) (Chong et al., 2018) was used for statistical analysis. All raw data are uploaded in Metabolights repository www.ebi.ac.uk/metabolights/MTBLS5514 (Haug et al., 2020) and treated data can be seen in supporting information.

Statistically significant metabolites were tentatively annotated by matching the obtained accurate m/z to those published in the selected databases, namely, KEGG (Hashimoto et al., 2006), HMDB (Wishart et al., 2022) and Metlin (Smith et al., 2005) within a mass accuracy window of 5 ppm. Finally, MS/MS analysis performed in the QC samples using MzCloud and MassBank as databases confirmed the identity of these compounds. Unequivocal identification was achieved by co-injection of compounds with available commercial standard solutions. The standards were prepared at concentrations of 25 ng/ml for their analysis in positive polarity and at 50 ng/ml for their analysis in negative polarity.

A pathway enrichment analysis was performed using Metaboanalyst version 5.0 with these annotated metabolites, using two metabolic databases, the Kyoto Encyclopedia of Genes and Genomes (KEGG), and the Small Molecule Pathway Database (SPMDB).

### 2.6 Western blot

Protein concentration was determined using Bradford Protein Assay Kit (BioRad, Hercules, CA, United States). Fifteen micrograms of protein samples were run on a resolving 10% acrylamide/polyacrylamide gel and transferred to a PVDF membrane. All primary antibodies (anti-PSMA diluted 1:1,000; anti-PRMT2 diluted 1:500; anti-PARP diluted 1:1,000; anti-PARG diluted 1:1,000; anti-CPT1alpha diluted 1:1,000; anti-PGC1alpha diluted 1:1,000; anti-βactin diluted 1:5,000) from Cell Signaling Technology (Danvers, MA, United States) were incubated at 4°C overnight. After washing, membranes were incubated with the secondary antibodies (1: 5,000 diluted horseradish peroxidase (HRP)-anti-mouse (Sigma-Aldrich (St. Louis, MO, United States) and 1:1,000 diluted anti-rabbit IgG (Cell Signaling Technology (Danvers, MA, United States). The bands were visualized by a chemiluminescence reagent (Cell Signaling Technology). Gel imaging Chemidoc MP System (BioRad, Hercules, CA, United States) was used to visualize and examine the protein bands. Bands were quantified using Scion Image 4.0 (Scion Corporation, Chicago, Illinois, United States).

### 2.7 RNA extraction and reverse transcription quantitative polymerase chain reaction

Cellular RNA was extracted from cells using the RNeasy Mini Kit (Qiagen, Hilden, Germany). Total RNA (2 µg) underwent cDNA synthesis using SuperScriptTM RT (Roche, Basel, Switzerland) according to the manufacturer’s protocol. qPCR was performed in a 10 µL volume using SYBR-Green PCR Master Mix (Takara Bio, Inc., Kusatsu, Japan) on a 7,500 Real-Time PCR System (Applied Biosystems Inc., Foster City, CA, United States). PCR amplification was carried out using the following primer sequences: Nanog-F 5′-TTT​GTG​GGC​CTG​AAG​AAA​C-3′, Nanog-R 5′-AGG​GCT​GTC​CTG​AAT​AAG​CAG-3'; Oct4-F 5′-GAC​AGG​GGG​AGG​GGA​GGA​GCT​AGG-3′, Oct4-R 5′-CTT​CCC​TCC​AAC​CAG​TTG​CCC​CAA​AC-3'; ABCB1A-F 5′-TTG​CTG​CTT​ACA​TTC​AGG​TTT​CA-3′, ABCB1A-R 5′-AGC​CTA​TCT​CCT​GTC​GCA​TTA-3'; AR-F 5′-CCA​GGG​ACC​ATG​TTT​TGC​C-3’; AR -R 5′-CGA​AGA​CGA​CAA​GAT​GGA​CAA-3’.

### 2.8 Fatty acid β-oxidation assay

Fatty acid β-oxidation was measured using assay kits from the Biomedical Research Service Center, State University of New York (Buffalo, NY, United States). Cells were lysed in 50 µL Cell Lysis Solution and centrifuged at 10,000 g at 4°C for 5 min. The supernatant is harvested and stored at -80°C. Protein concentration was assessed by Bradford assay and normalized to 1 mg/ml. Twenty microliters of the samples were added in duplicate to a 96-well plate and 50 µl of FAO Assay Solution or control solution was added to each sample. The plate was incubated in a non-CO2 incubator at 37°C for 60 min. All experiments were terminated by adding 50 µl of 3% acetic acid, and the plate was read at a wavelength of 492 nm with a microplate reader (iMARK, Bio-Rad Laboratories, Inc., Hercules, CA, United States). Control well reading was subtracted from reaction well reading for each sample and FAO activity was calculated in IU/L = µmol/(L•min) = ΔO.D. × 1,000 × 70 µl/(30 min × 0.5 cm × 18 × 20 µl) = ΔO.D. × 12.96. Enzyme activity was presented as units/µg proteins.

### 2.9 Statistical analysis

Statistical significance in qPCR and wester blotting assays, was estimated with Graphpad 9.0 (La Jolla, CA, United States) software using 2-way ANOVA and Tukey’s or Sidak’s multiple comparison test when indicated. Data are presented as the mean ± SD.

## 3 Results

### 3.1 The cell line LN-Flu is resistant to 2-hydroxyflutamide and expresses stem cell markers

**FIGURE 1:**
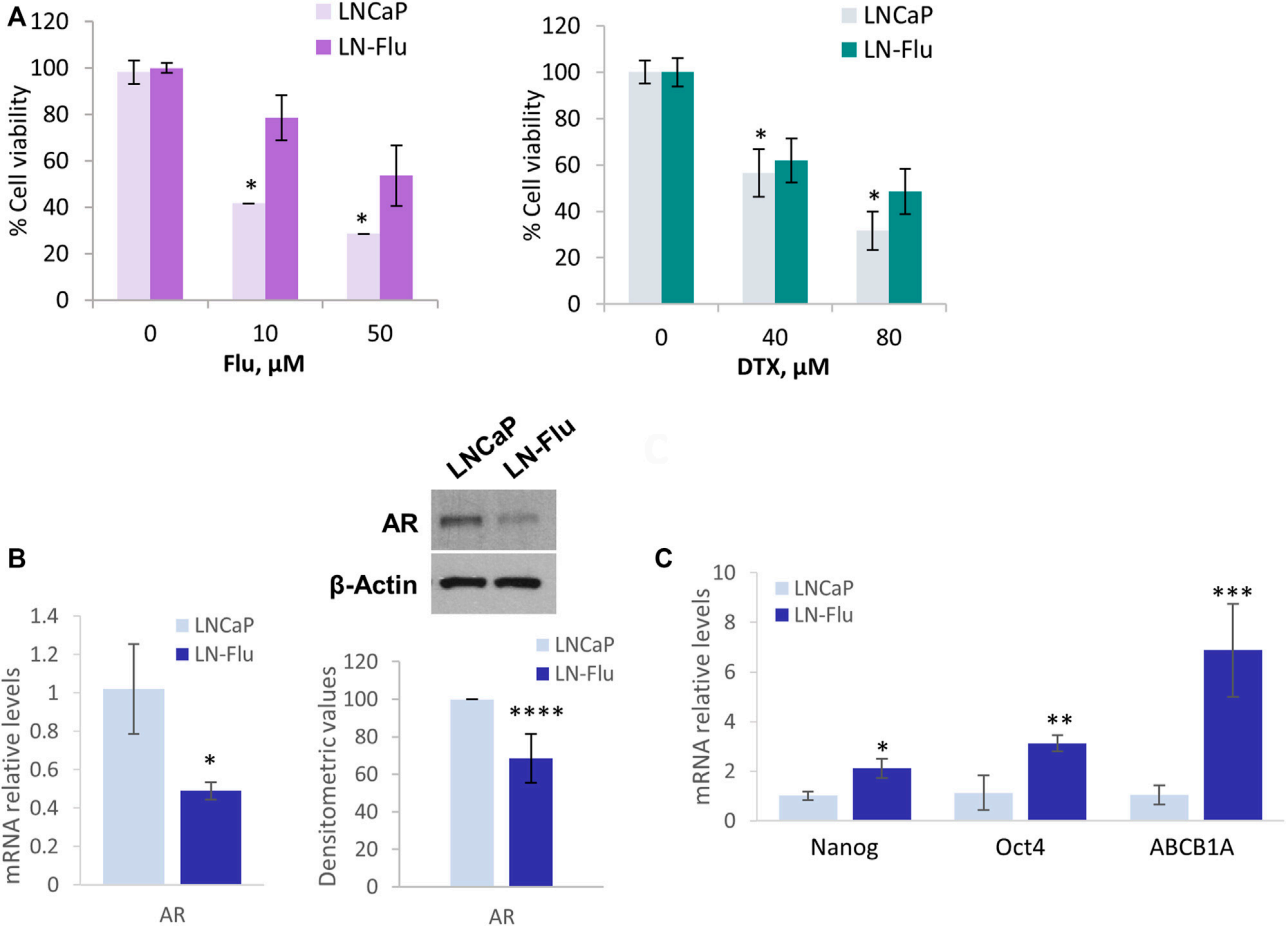
LN-Flu cells exhibit chemotherapy resistance and express stem cell markers. LNCaP cells were adapted for six months to grow in the presence of 2 µm 2-hydroxyflutamide after which they were renamed LN-Flu. **(A)** MTT Cell viability assay of LNCaP and LN-Flu cells. Cells (1.5 × 10^5^cells/well) were seeded into 12-well plates and treated with the indicated doses of 2-hydroxyflutamide or docetaxel for 24 h. 100 μl of MTT [3-(4, 5-dimethyl-2-thiazolyl)-2, 5-diphenyl-2H-tetrazolium bromide] dye solution was added to each well and incubated at 37°C for 1 h. Subsequently, the cells were lysed with 2-propanol and the optical density was measured at 595 nm. Cell viability was calculated as the percentage compared to the control cells, which were arbitrarily assigned 100% viability. **(B)** Levels of AR expression in each cell line, determined by Western blot and qPCR. **(C)** Levels of stem cell markers expression determined by qPCR. Results are the mean ± S.D. of three independent experiments. *p < 0.05 and **p < 0.01 significant difference between LNCaP and LN-Flu cells by two-way ANOVA and Sidak’s multiple comparisons test.

To study metabolic changes related to cancer stem cells and therapy resistance we developed a cell line resistant to the androgen receptor and chemotherapeutic 2-hydroxyflutamide. To this end, prostate cancer LNCaP cells were slowly adapted to growth in the presence of 2-hydroxyflutamide, after which they developed resistance to the antiandrogen as well as were less sensitive to the antimitotic docetaxel (Sanchez et al., 2020). To corroborate the chemoresistance in the cells used in this study, we assessed the effect of the IC50 and IC75 doses of docetaxel and 2-hydroxyflutamide on cell viability. MTT viability assay shows that 10 µM 2-hydroxyflutamide at 24h, reduced LNCaP cell viability to 40% whereas in LN-Flu cells only reduced cell viability to 83% (Figure 1A) which is in line with previous reports (Sanchez et al., 2020). Likewise, 40 µM docetaxel reduced LNCaP cell viability to 50% but only to 80% in LN-Flu cells (Figure 1A). Western blot and qPCR analysis revealed that LN-Flu cells decreased the expression of the androgen receptor (Figure 1B). Interestingly, LN-Flu cells had increased expression of the typical pluripotent genes Nanog and Oct4 as well as of the transporter ABCB1A or glycoprotein P, all of them related to the acquisition of stem cell features and resistance to chemotherapy (Figure 1C). These results indicate that LN-Flu cells are cancer stem-like cells and display resistance to the androgen receptor antagonist and therefore is a good model to analyze the metabolome of prostate cancer drug-resistant cells.

### 3.2 Metabolic fingerprinting on stem cell samples by LC–HRMS

**FIGURE 2:**
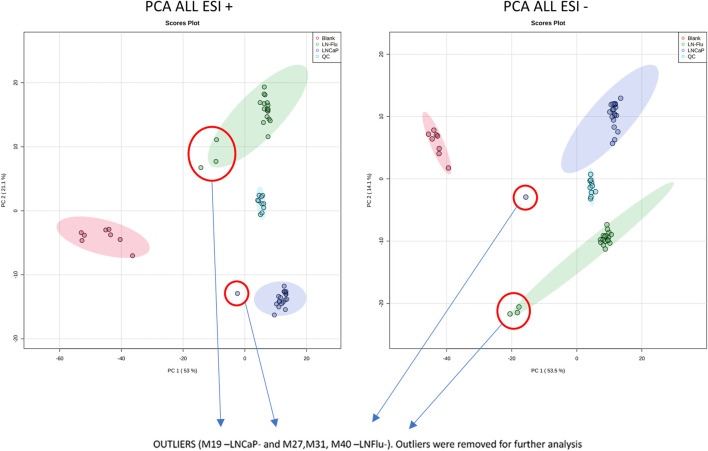
The Principal Component Analysis of metabolomic data from all samples in both positive and negative MS ionization methods shows good separation between group classes. Blank samples (red dots) are perfectly separated from the rest, and QC samples (light blue dots) are placed in between LNFlu (green dots) and LNCaP (dark blue dots) samples showing good consistency in the metabolomic study.

Using the above explained LC-HRMS method, metabolic fingerprints of cell cultures from the LNCaP group and the LN-Flu group were obtained. Cells were seeded and after 72 h, cell extracts were obtained, as detailed in the methods section, for the metabolomic study. Compound Discoverer data processing and the subsequent peak filtering process allowed us to detect 850 features in ESI + mode, and 446 in ESI- mode in all the 40 samples (see www.ebi.ac.uk/metabolights/MTBLS5514). The resulting data matrix with the individual values of peak area from each feature in every sample was then submitted to statistical analysis. The overall differences between the LNCaP group and the LN-Flu group were first evaluated by PCA. A clear separation was observed between both groups in both ionization methods (see Figure 2) evidencing relevant metabolic differences between both cell cultures. The principal component 1 (PC1) could explain 53% of the total variance in ESI+ and 53.5% in ESI-. However, the main separation axis between both groups is along principal component 2 (PC2), which explains 21.1% of the variability in ESI+ and 14.1% in ESI-. This suggests that although some differences can be found, the vast majority of the metabolome is not altered between both groups, as expected in an untargeted metabolomic approach. Furthermore, the PCA analysis was used to ensure the quality of the analysis. As can be seen, QC samples are placed between both groups in both PC1 and PC2, ensuring a good analytical quality. Also, some samples were deemed as outliers, as they appear outside of the 95% confidence interval and were therefore removed from further statistical analysis. These samples are M19 in LNCaP group, and M27, M31 and M40 from LN-Flu group.

**FIGURE 3:**
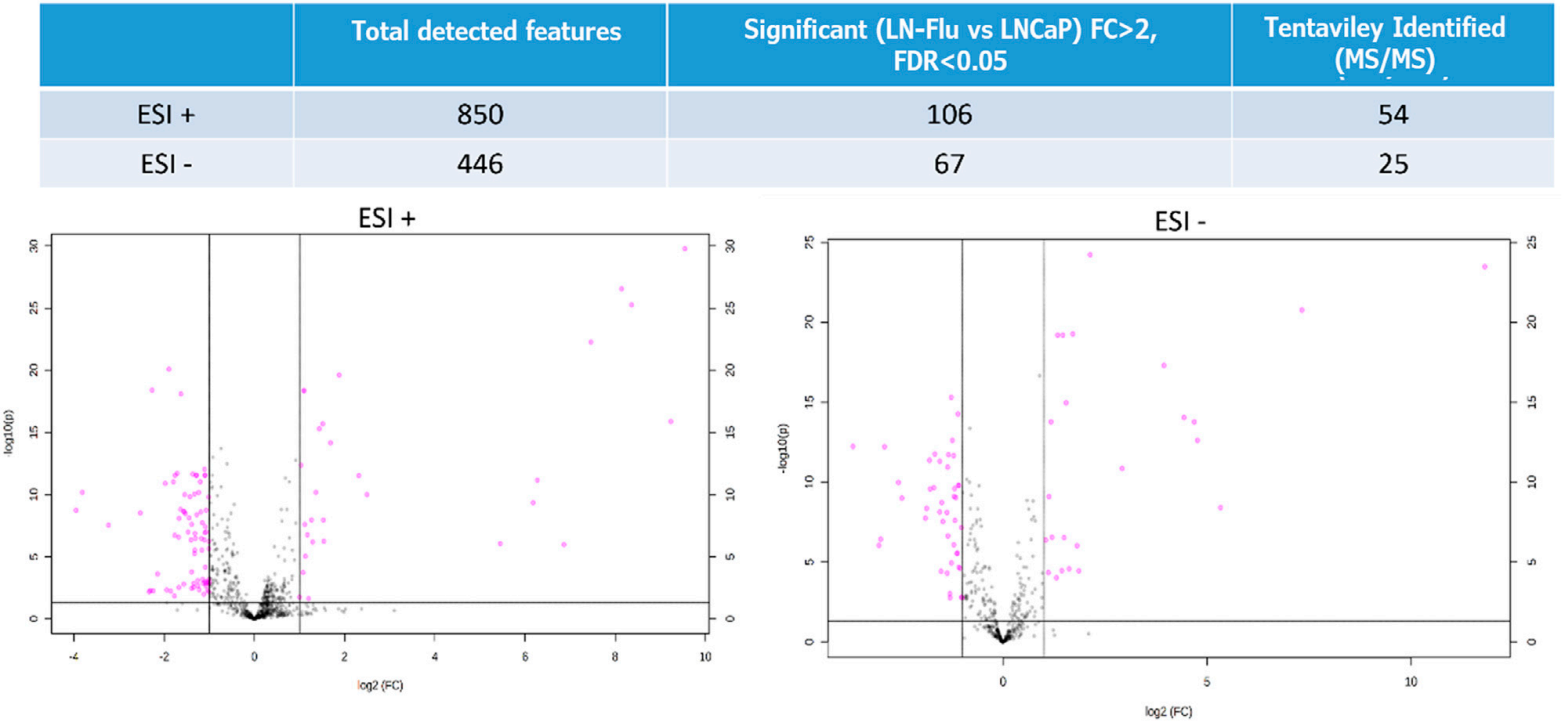
Volcano Plot showing the metabolomics statistically significant features in the LNFlu vs. LNCaP comparison, using both positive and negative MS ionization methods. Black dots represent non significant features detected in the study, and purple dots represent significant features in the LNFlu vs. LNCaP comparison (FDR <0.05 and FC > 2 or FC < 1/2).

After ensuring data quality with the PCA, a ShapiroWilk’s test was used to check the normal distribution of the data, and a Volcano Plot was used to evaluate statistically significant differences between both groups. From this test, 106 out of the 850 metabolites in ESI+ and 67 out of 446 in ESI- showed significant differences (FDR <0.05 and FC > 2 or FC < 1/2) between LNCaP and LN-Flu groups (Figure 3). Among them, 28 features showed significantly higher MS response in the LN-Flu group, while 78 features showed the opposite behavior in ESI+. In ESI-, 24 features presented higher concentration in LN-Flu group, while 43 had lower concentrations (see Supporting Information, Supplementary Table S2). Out of these statistically significant features, 54 were tentatively annotated using MS/MS data in ESI + mode, while 25 were annotated in ESI-. The unequivocal identification of these metabolites using commercially available standards was achieved for 14 metabolites in positive ionization mode and 10 in negative ionization (Table 1).

**TABLE 1:** Significant metabolites (FDR < 0.05 and FC > 2 or FC < 1/2) unequivocally identified.

ESI polarity	ID	Annotated name	FDR	FC (LN-Flu/LNCaP)	Regulation
ESI+	**ID_241**	trans-3-Indoleacrylic acid	2.20E-12	0.388	Down LN-Flu
	**ID_70**	Octanoylcarnitine	2.77E-12	0.467	Down LN-Flu
	**ID_222**	Spermidine	1.59E-10	0.495	Down LN-Flu
	**ID_842**	N-Acetylaspartylglutamic acid	8.21E-09	0.314	Down LN-Flu
	**ID_144**	Aspartic acid	1.83E-08	0.449	Down LN-Flu
	**ID_183**	N-Acetyl-L-aspartic acid	3.92E-08	0.470	Down LN-Flu
	**ID_846**	Valine	1.35E-07	0.401	Down LN-Flu
	**ID_155**	Phenylalanine	3.37E-07	0.443	Down LN-Flu
	**ID_58**	Methionine sulfoxide	1.04E-03	0.468	Down LN-Flu
	**ID_146**	Cysteinyl glycine (Cys-Gly)	6.56E-03	0.497	Down LN-Flu
	**ID_176**	N3, N4-Dimethyl-L-arginine SDMA	2.43E-20	3.681	Up LN-Flu
	**ID_114**	Proline	4.28E-19	2.143	Up LN-Flu
	**ID_152**	Histidine	6.60E-11	2.573	Up LN-Flu
	**ID_280**	ADP-ribose	9.19E-06	2.188	Up LN-Flu
ESI-	**ID_357**	Tryptophan	4.88E-16	0.415	Down LN-Flu
	**ID_342**	Acetyl-CoA	5.75E-13	0.078	Down LN-Flu
	**ID_322**	Phenylalanine	1.91E-12	0.396	Down LN-Flu
	**ID_315**	Coenzyme A	6.09E-13	0.134	Down LN-Flu
	**ID_152**	5-Aminovaleric acid	9.12E-10	0.450	Down LN-Flu
	**ID_246**	S-Nitroso-L-glutathione (GSNO)	7.37E-09	0.342	Down LN-Flu
	**ID_545**	Lauric acid (dodecanoic acid)	6.92E-08	0.494	Down LN-Flu
	**ID_95**	Histidine	6.37E-20	2.762	Up LN-Flu
	**ID_175**	Glutamyl alanine	2.79E-07	2.299	Up LN-Flu
	**ID_242**	ADP-ribose	4.17E-07	2.068	Up LN-Flu

ESi polarity: ionization mode (positive or negative) in the MS; ID: automatic ID given to each feature in the metabolomics analysis, before the identification; Annotated name: identified metabolites; FDR: False Discovery Rate; FC (LN-Flu/LNCaP): Fold change of the comparison LN-Flu versus LNCaP; Regulation: up or downregulation of the metabolite in LN-Flu versus LNCaP.

**FIGURE 4:**
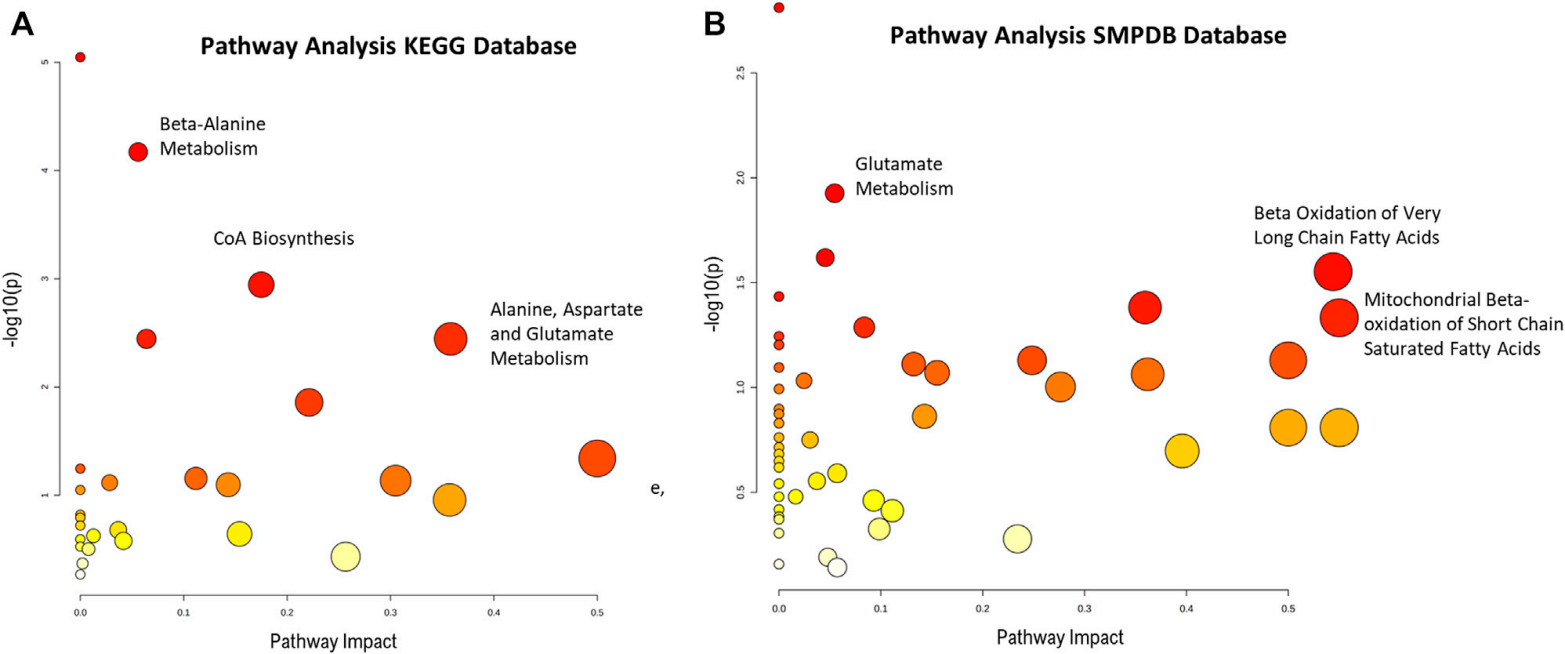
Pathway enrichment analysis from absolutely identified metabolites in **(A)** KEGG and **(B)** SPMDB databases. Graph shows the impact the statistically significant identified metabolites have in the different pathways (x-axis) together with the statistical significance of those metabolites (y-axis). Highlighted pathways have several statistically significant metabolites that in turn produce a high impact in the pathway.

Finally, a pathway analysis performed with the unequivocally identified metabolites, revealed several pathways that were significantly impacted by these metabolites (Figure 4). These pathways include energy metabolism such as fatty acid degradation and amino acid metabolism.

### 3.3 Biological relevance of the significant metabolites

To validate the results obtained in the metabolomic study, and to examine the biological relevance in chemotherapy-resistant prostate cancer stem cells of the most significant metabolites described before, we examined the levels of the main enzymes involved in the metabolic pathways highlighted by the KEGG and SMPB analyses.

#### 3.3.1 Fatty acid oxidation

**FIGURE 5:**
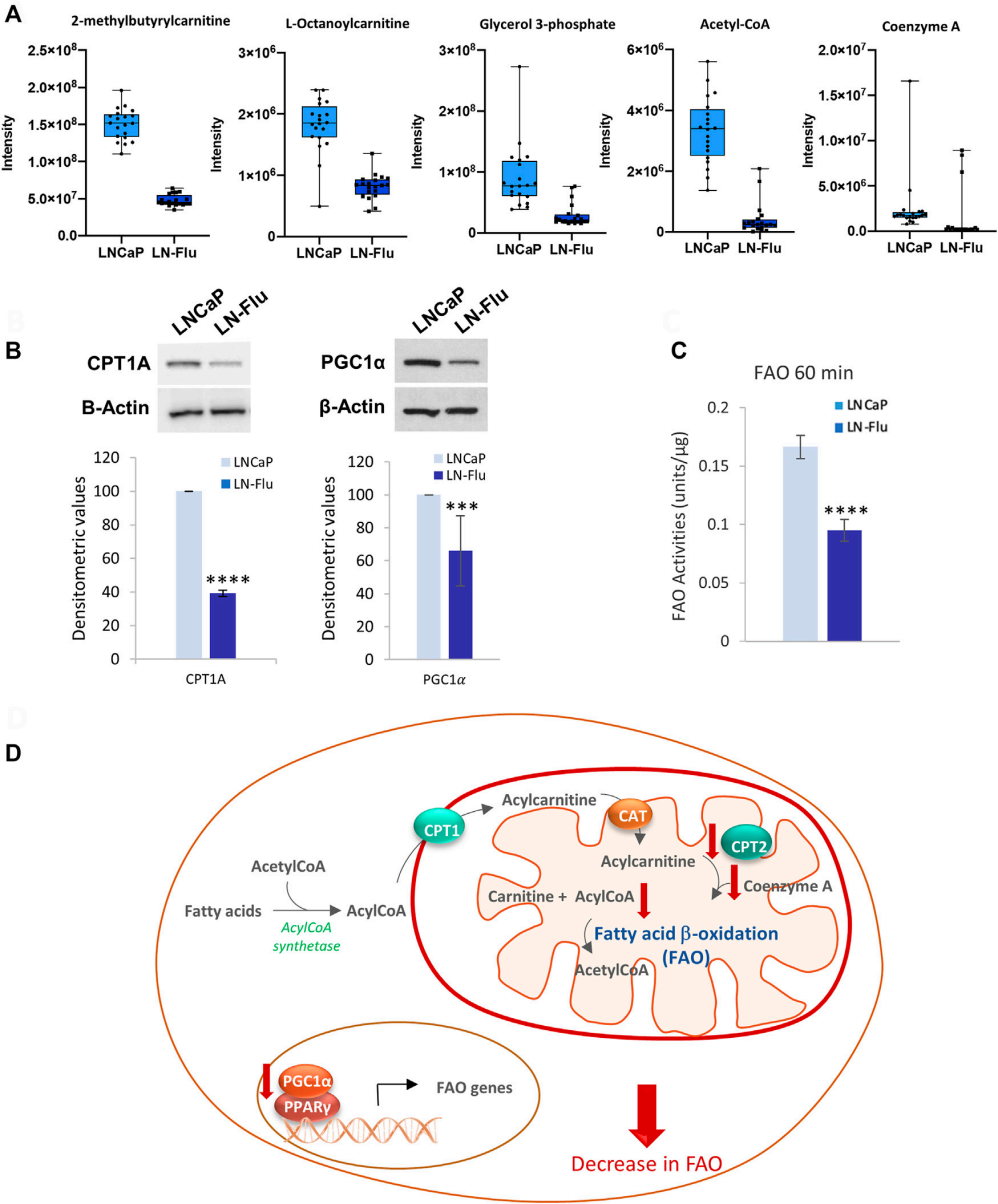
Fatty acid β-oxidation in prostate LN-Flu cells. **(A)** Box and whisker plot of metabolites involved in the pathway. **(B)** Levels of carnitine palmitoyl transferase 1 (CPT1) and Peroxisome proliferator-activated receptor-gamma coactivator 1α (PGC1α) determined by Western blot. Densitometric analysis of the western blot bands from three independent experiments is shown on the right. **(C)** Fatty acid β-oxidation determined by Results are the mean ± S.D. of three independent experiments. **p < 0.01 significant difference between LNCaP and LN-Flu cells by two-way ANOVA and Sidak’s multiple comparisons test. **(D)** Fatty acid β-oxidation (FAO) scheme showing the metabolites and enzymes we have found up (red arrow up) or down (red arrow down) in LNFlu cells compared to LNCaP cells. CPT1, carnitine palmitoyltransferase 1; CAT carnitine:acylcarnitine translocase; CPT2, carnitine palmitoyltransferase 2; PGC1α, peroxisome proliferator-activated receptor-gamma coactivator 1α; PPARγ, peroxisome proliferator-activated receptor γ.

Fatty acids oxidate by the β-oxidation pathway (FAO) in the mitochondria to yield acetyl-CoA which enters the TCA cycle to fuel oxidative phosphorylation. Fatty acids are transported into the inner mitochondrial membrane by conjugation of long-chain fatty acids to carnitine forming acylcarnitine. In addition, acetyl-CoA also plays a role in protein acetylation. Since the metabolome analysis showed a decrease in coenzyme A and acyl-CoA as well as several acylcarnitines (Figure 5A), and impact of the beta-oxidation of long and short fatty acids (Figure 4), we evaluated the level of carnitine palmitoyltransferase 1A (CPT1A), which is responsible for mitochondrial β-oxidation by transporting fatty acids into mitochondria and FAO in the prostate cells. As shown in Figure 5B, the levels of CPT1A were strongly decreased in the cancer stem-like cells LN-Flu. Likewise, the expression of the peroxisome proliferator-activated receptor-gamma coactivator 1α (PGC1α), an essential factor involved in mitochondrial energy homeostasis and biogenesis, was notably decreased in LN-Flu cells (Figure 5B), suggesting a decrease in mitochondrial function. Therefore, to corroborate whether the drug-resistant cancer stem-like LN-Flu cells showed slower fatty acid β-oxidation, we measured the oxidation of fatty acids using octanoyl-CoA as substrate as described in the methods section. According to their lower levels of acyl CoA, Coenzyme A, CPT1, and PGC1α, FAO of LN-Flu cells was notably decreased (Figure 5C). Altogether, these results indicate that the catabolism of fatty acid to obtain energy is diminished in the cancer stem-like cells pointing to a metabolic dormancy state (Figure 5D).

#### 3.3.2 Glutamate, N-Acetylaspartate metabolism

**FIGURE 6:**
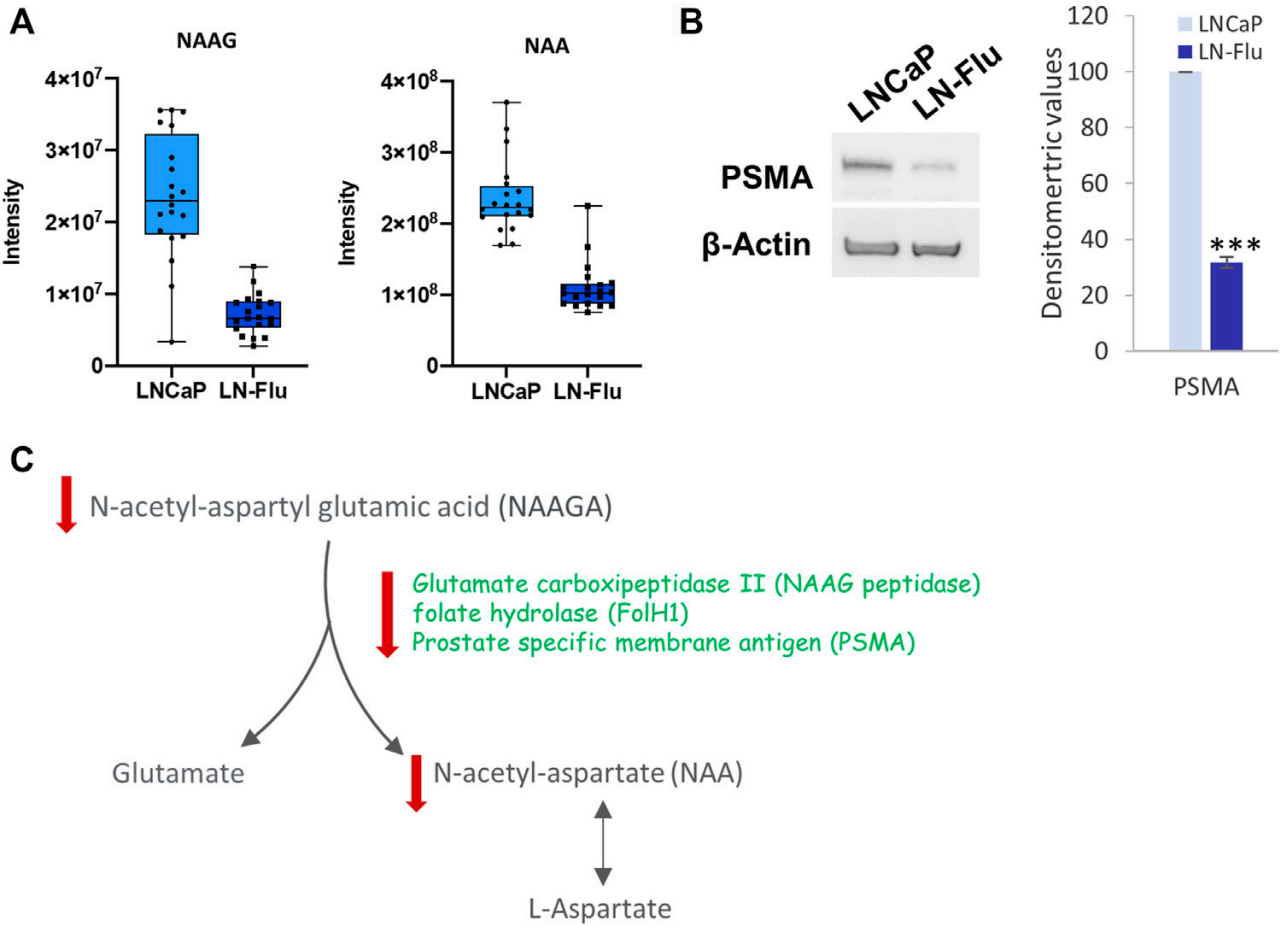
N-acetyl aspartate biosynthesis in prostate LN-Flu cells. **(A)** Box and whisker plot of metabolites involved in the pathway. **(B)** Levels Prostate specific membrane antigen (PSMA) determined by Western blot. Densitometric analysis of the western blot bands from three independent experiments is shown on the right. Results are the mean ± S.D. of three independent experiments. ***p < 0.005 significant difference between LNCaP and LN-Flu cells by two-way ANOVA and Sidak’s multiple comparisons test. **(C)** N-acetyl-aspartate (NAA) biosynthesis showing the metabolites and enzymes we have found up (red arrow up) or down (red arrow down) in LNFlu cells compared to LNCaP cells.

The metabolomic analysis showed that both NAA and its precursor N-acetyl-aspartyl glutamic acid (NAAGA), were decreased in the cancer stem-like LN-Flu cells (Figure 6A) suggesting a depletion of this pathway. This notion was confirmed by the surprisingly low levels of PSMA detected in LN-Flu cells (Figure 6B). Aberrant N-acetylaspartate (NAA) concentrations have been detected in many pathological conditions including neurodegenerative disorders, obesity and type 2 Diabetes (Daniele et al., 2020). Although it is believed that it is only synthetized in brain, the prostate expresses the NAA synthetizing enzyme Glutamate carboxipeptidase II (NAAG peptidase) also named folate hydrolase (FolH1) and prostate specific membrane antigen (PSMA), which increases to the point of being considered one of the best tumor markers for prostate cancer (Kaewput and Vinjamuri, 2022; Schoder et al., 2022). In addition, NAA promotes oxidative stress and stimulates lipid peroxidation and protein oxidation (Surendran and Bhatnagar, 2011). Our results indicate that this pathway is downregulated in the resistant LN-Flu cells compared to the sensitive parental LNCaP cells (Figure 6C).

#### 3.3.3 Methionine, spermidine and glutathione

**FIGURE 7:**
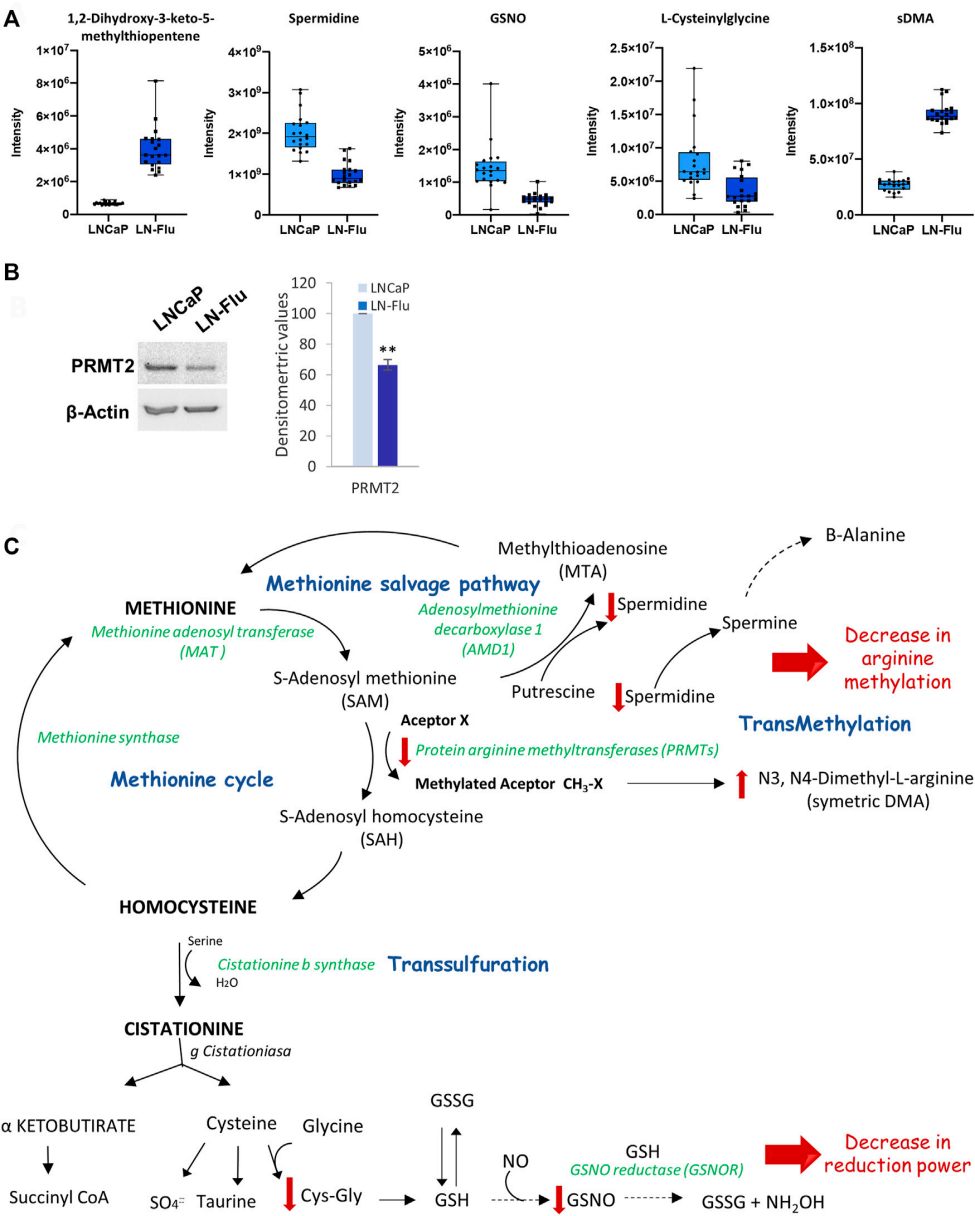
Methionine metabolism and methylation in prostate LN-Flu cells. **(A)** Box and whisker plot of metabolites involved in the pathway. **(B)** Levels of Protein arginine methyltransferase 2 (PRMT2) determined by Western blot. Densitometric analysis of the western blot bands from three independent experiments is shown on the right. Results are the mean ± S.D. of three independent experiments. **p < 0.01 significant difference between LNCaP and LN-Flu cells by two-way ANOVA and Sidak’s multiple comparisons test. **(C)** Methionine metabolism (Methionine cycle, methionine salvage pathway and transsulfuration) showing the metabolites and enzymes we have found up (red arrow up) or down (red arrow down) in LN-Flu cells compared to LNCaP cells.

To contextualize the increase of N3, N4-Dimethyl-L-arginine (sDMA), and the decrease of spermidine observed in the metabolomic analysis (Figure 7A), we determined the levels of the main enzyme responsible for methylation reactions PRMT2. As observed in Figure 7B, levels of PRMT2 were decreased in LN-Flu cells compared to LNCaP cells. Within the cell, methionine is recycled from homocysteine by methionine synthase, *via* the methionine cycle, which is linked to nutrient status through one-carbon metabolism. In addition, *via* the methionine salvage pathway, methionine yields S-adenosylmethionine (SAM) which plays an essential part in methyl donation for methylation reactions catalyzed by protein arginine methyltransferases (PRMTs). This pathway is essential for gene expression *via* DNA methyltransferases (DNMT) and histone methyltransferases, lipid metabolism, and polyamine synthesis (Figure 7C). Additionally, methionine can also be recycled from the SAM-dependent polyamine biosynthesis by-product methylthioadenosine (MTA), which is further processed by the enzyme methylthioadenosine phosphorylase (MTAP) *via* the methionine salvage pathway (Figure 7C) which generates spermidine, a polyamine which interacts with negatively charged macromolecules regulating cell growth, differentiation, and apoptosis (Holbert et al., 2022). Our results indicate that arginine methylation as well as the methionine salvage pathway are decreased in the stem-like cells. In addition, methionine metabolism is connected with glutathione (GSH) synthesis, an antioxidant tripeptide that protects against oxidant injury. GSNO can be formed from NO and GSH through the reduction of ferric cytochrome C providing a link between GSNO formation and the cellular redox status (Broniowska et al., 2013). Therefore, our results showing a depletion of L-cysteinyl glycine and S-Nitroso-L-Glutathione (GSNO) indicate a decrease in the cell reduction power (Figure 7C).

#### 3.3.4 ADP-ribose

**FIGURE 8:**
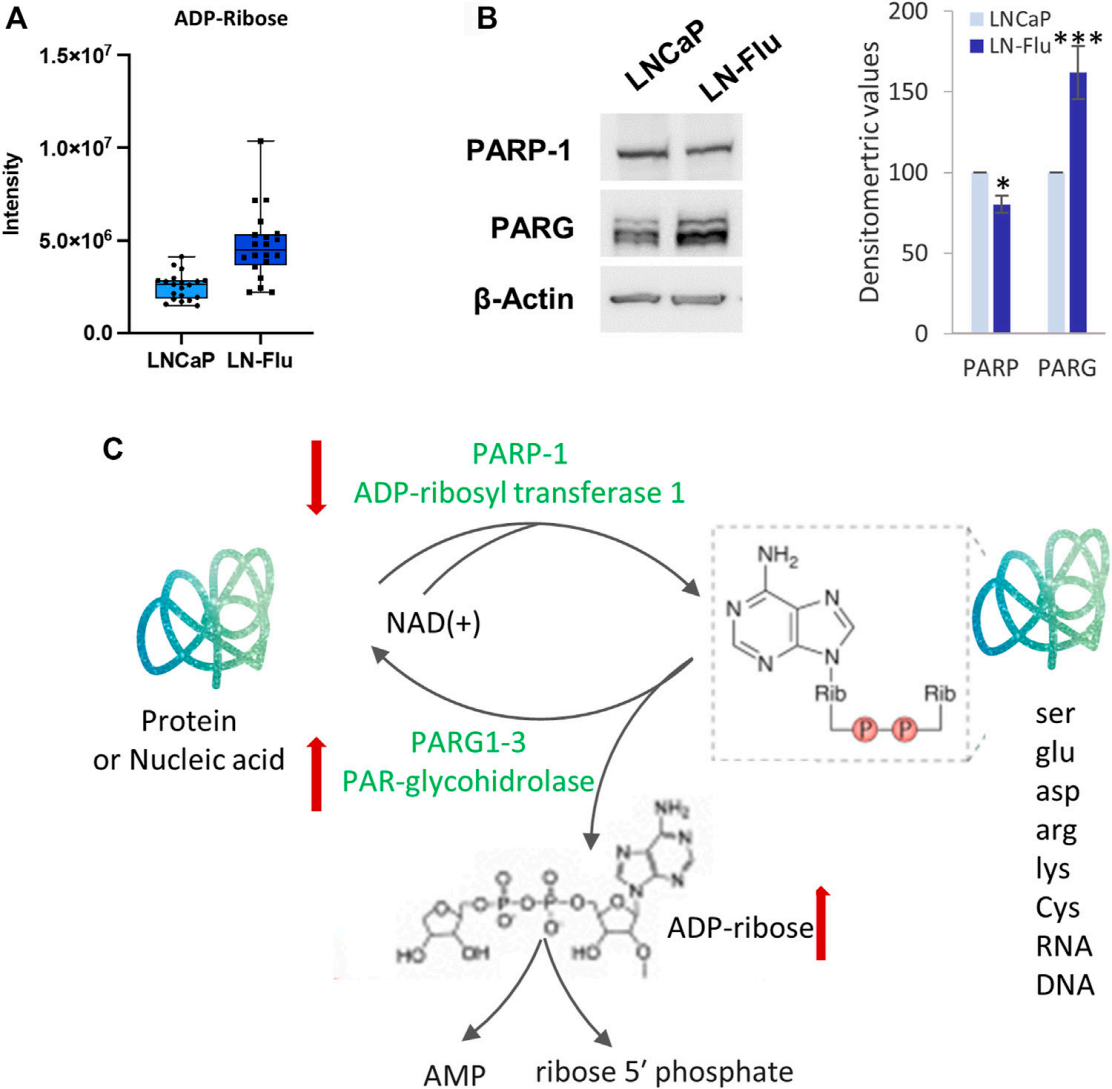
ADP-ribosylation is modified in the drug-resistant cancer stem cells LN-Flu. **(A)** Box and whisker plot of metabolites involved in the pathway. **(B)** Levels of ADP-ribosyl transferase 1 (PARP-1) and PAR-glycohydrolase (PARG1-3) determined by Western blot. Densitometric analysis of the western blot bands from three independent experiments is shown on the right. Results are the mean ± S.D. of three independent experiments. *p < 0.05 and **p < 0.01 significant difference between LNCaP and LN-Flu cells by two-way ANOVA and Sidak’s multiple comparisons test. **(C)** ADP-ribosylation reaction showing the metabolites and enzymes we have found up (red arrow up) or down (red arrow down) in LN-Flu cells compared to LNCaP cells.

Finally, an enrichment of intracellular ADP-ribose was also observed in the LN-Flu cells (Figure 8A). To analyze the enzymes involved in ADP-ribose metabolism we examined the levels of PARP-1, the founding member of the PARP family and the most extensively studied, and PAR-glycohidrolases 1–3 (PARG1-3) which remove ADP-ribosyl modification yielding free ADP-ribose. As shown in Figure 8B, levels of PARP-1 were sightly but significantly decreased whereas those of PARG1-3 were increased in the stem-like resistant LN-Flu cells compared to LNCaP cells. ADP-ribose is involved in ADP-ribosylation, a post-translational modification that regulates the activity of many proteins involved in key processes like DNA damage repair, cell proliferation and differentiation, metabolism, stress, and immune responses (Navas and Carnero, 2021). ADP-ribose is added consecutively to the molecules by PAR polymerases (PARPs) from NAD + resulting in the poly-ADP-ribosylation of the target molecules (Figure 8C). Our results showing a decrease of PARP-1 and an increase of PARG1-3 correlate with the increased ADP-ribose found in the metabolomic analysis and indicate a decrease in the ADP-ribosylation of molecules.

## 4 Discussion

Chemoresistance is one of the most challenging difficulties in prostate cancer treatment. The high prevalence of chemoresistant cancer makes it urgent to deepen our understanding of chemoresistance mechanisms and to develop novel therapeutic strategies. Among the mechanisms underlying chemoresistance stand out the existence of cancer stem cells with unique properties including high multi-drug resistance transporters expression, and self-renewal ability that sustain the tumor even in the presence of chemotherapeutics. However, while metabolic reprogramming is nowadays considered a hallmark of cancer (Fouad and Aanei, 2017), little is known about the metabolic pathways underlying the plastic nature of CSCs, which are capable of residing in a dormant state, and able to rapidly proliferate when the need to repopulate the tumor mass arises (Mancini et al., 2018).

Cancer stem cells are an important subpopulation in prostate tumors and are mostly responsible for antiandrogen therapy resistance. In a recent epigenetic and transcriptomic study of castration-resistant prostate cancer patients, Tang et al. (Tang et al., 2022) identified four subgroups according to their lineage plasticity and transcription factors involved. They found a stem cell-like (SCL) subtype which was the second most prevalent group and was associated with a poorer response to the new generation of androgen receptor inhibitors. Interestingly, those SCL tumors were negative or had low expression of the AR (Tang et al., 2022). In another study, Han et al. identified a stem-like PCa subtype of metastatic castration-resistant prostate cancer that arose from AR-positive cells as a consequence of its blockade with enzalutamide and therapy-induced lineage plasticity (Han et al., 2022). In this study, we have used a prostate cancer cell line adapted to grow in the presence of the antiandrogen 2-hydroxyflutamide, that displays characteristics of stem cells like an enhanced expression of the pump that efflux accumulated drugs inside the cell, ABCB1A, increased expression of the pluripotency factors Nanog and the Yamanaka factor Oct4, as well as low levels of the AR. Remarkably, those cells exhibited less sensitivity to the chemotherapeutics flutamide as well as docetaxel and therefore are a good model to study the metabolic changes related to cancer chemotherapy resistance.

Accumulating evidence suggests that lipid and amino acid metabolism alteration is closely related to drug resistance in tumors (Yang et al., 2022). It has been recently reported that metastatic ovarian cancer stem cells display different lipid patterns than their primary tumors. Those cells depend on lipophagy for the utilization of lipids rather than the conventional lipolytic pathway and accumulate numerous cytoplasmic lipid droplets and lipophagic vesicles in contrast to their primary tumors (Sandeep et al., 2022). In the same line, hematopoietic stem cells depend on mitochondrial fatty acid β-oxidation to regulate differentiation. Cells that self-renew in a symmetrical division had higher levels of proteins involved in FAO whereas cells that differentiate in an asymmetrical division had lower levels (Ito et al., 2016). Our results showed a decrease in fatty acid β-oxidation in the cancer resistant stem-like cells compared to their parental cells suggesting that they are probably undergoing asymmetrical divisions to maintain proliferating tumor cells.

Cancer cells distinguish for their high required levels of methionine, a fact known as methionine addiction or Hoffman effect, as it was first described by Hoffmann (Hoffman, 1985; Hoffman et al., 2019). Human pluripotent stem cells also use high levels of methionine for protein synthesis and methylation reactions involved in epigenetic regulation. Interestingly, methionine and SAM are key factors regulating pluripotency and differentiation. Methionine deprivation induces a decrease in SAM levels triggering epigenetic changes that modulate the expression of genes involved in pluripotency (Shiraki et al., 2014) and maintains cells in a quiescent state (Homma et al., 2022). Our results show a depletion of the arginine methyl transferase PRMT2 and a rise in sDMA suggesting that arginine methylation is reduced in the resistant cells. This is in good agreement of previous results describing that PRMT inhibition trigger Oct4-dependent reprogramming of embryonic fibroblasts implying that inhibition of protein arginine methylation might be the inductor of the reprogramming process (Yuan et al., 2011).

Altogether, our results indicate that the resistant prostate cancer cells are metabolically dormant, showing repression fatty acid oxidation, methionine, and ADP-ribosylation pathways. According to previous hypothesis (Shyh-Chang and Ng, 2017), it seems that long-term self-renewing stem cells maintain their multipotent capacity in a hypoxic environment and downregulate mitochondrial activity (Han et al., 2018). Moreover, it has been suggested that in stress conditions cancer cells can suffer a phenotypic transition from a proliferating state to a dormant state characterized by no proliferation, no death, no senescence, resistance to chemotherapy, high expression of dormant markers, metabolic suppression, and recovery to active status (Morales-Valencia and David, 2022). In fact, tumor dormancy is a critical stage in cancer development where cancer cells can remain occult, asymptomatic, and resistant to therapy. Tumor dormant cells limit the efficacy of chemotherapy which is mostly directed towards highly dividing cells, driving recurrence and drug resistance. The capacity of tumor cells to stay dormant and reappear dramatically with a more aggressive performance resistant to chemotherapy is characteristic of prostate cancer (Cackowski and Heath, 2022). Despite the importance of tumor dormancy in chemotherapy resistance of prostate cancer, the mechanisms underlying dormancy entry and the metabolic pathways involved remain largely unexplored. In this study, we have explored the metabolic adaptation of prostate cancer cells resistant to chemotherapy. Recent findings indicate that overexpression of Nanog is associated with prostate cancer cells dormancy (Cackowski et al., 2017). Likewise, a previous study by Zhang et al. demonstrated that Nanog overexpression induced dormancy of colorectal cancer cells (Zhang et al., 2022). Accordingly, our results show that chemoresistance is associated with an overexpression of Nanog and a metabolic switch off. These results shed light on the pathways underpinning the entry of cancer cells into dormancy. Then, a strategy to overcome chemoresistance could be to induce dormancy escape by rewiring the metabolic pathways that remain downregulated in the resistant cells.

One of the major limitations in metabolomics is the identification of the unknown metabolites. Identification of metabolites is usually not a successful task and often results in the unequivocal identification of only a few metabolites. There are two reasons for this issue, the prohibitive costs or the non-availability of most of the standards, and the limited information that can be found in currently available databases. Another strong limitation is the lack of an analytical platform enabling the total coverage of the metabolome due to the heterogeneity of the broad spectrum of metabolites that can be present in the analyzed samples and the difference in the abundance of these metabolites as they might range from pmol to mmol (nine orders of magnitude). On the other hand, another limitation of the study is that we have used a unique resistant cell line. Although this has allowed the homogeneity of the samples to achieve better accuracy in the metabolomic analysis, the work might gain additional relevance using resistant cell lines to another different chemotherapeutics.

## Data Availability

The datasets presented in this study can be found in online repositories. The names of the repository/repositories and accession number(s) can be found below: www.ebi.ac.uk/metabolights/MTBLS5514
